# A Case of Bright’s Disease

**Published:** 1885-09-20

**Authors:** P. R. Cortelyou

**Affiliations:** Georgia


### A CASE OF BRIGHT'S DISEASE.—RECOVERY.

BY P. R. CORTELYOU, OF GEORGIA.

Miss M——, aged twelve years, contracted scarlatina in the spring of 1879, and, on the fifth day, feeling so well, was allowe to play on the floor ; as a consequence, was sick for three weeks with inflammation and suppuration of the outer ear. In the spring of 1880 she had a similar attack of the ear trouble, but less severe. In May, 1881, she had a slight attack of measles. February, 1883, there was a return of the ear trouble, with œdema of the face and extremities, which was noticed for the first time. Prior to this no trouble had been suspected, and there had been no examination of the urine.

In March, 1883, Dr. E. G. January, of New York City, saw the patient, and the condition was as follows : Marked œdema of face and lower extremities, with evidence or hypertrophied left ventricle of heart. The ophthalmascope showed no change in the fundus of the eye. The urine was found to be highly albuminous, with many casts, epitelial and blood. The patient was then sent to Fortress Monroe, and placed under care of Dr. Charles Page, U. S. A. The treatment consisted of rest in bed, tincture of iron, and milk diet.

May 9th, patient returned to Milwaukee, her home, and was under the care of Dr. Farnham. The general appearances and symptoms were same as when in New York. Patient was kept in bed ; absolute milk diet enforced ; skin and bowels carefully watched. The iron was continued, and gallic acid also given. In one month she was allowed to get up, and go about the house, and then out of doors. She had three attacks of vertigo at this time, and had suffered some in that respect for nearly one year. Three months daily examination of urine gave the following results: Lowest amount passed in twenty-four hours, 37 ounces; highest, 86 ozs.; specific gravity, 1005 to 1008. The amount of albumen decreased from 5 grammes to 0.07 of gramme in twenty-four hours, but was never absent. Microscope showed renal epithelium in fatty degeneration, granular and hyaline casts, and some fat casts, with occasional pus cells. August 24 patient weighed 77 pounds—a gain of 10 pounds since June.

October 31, urine still contained albumen, with small and large hyaline casts. The case at this time was considered by Drs. January, Page, and Farnham to be the large white kidnies, with fatty degeneration advanced to partial atrophy.

November 27, 1883, the patient first came under my care, having come to Marietta to escape the severe winter weather of Milwaukee. Upon examination, found the patient to have peculiar waxy color of skin, with some fulness under eyes; muscles soft, and inclined to be fatty; patient easily fatigued; urine, sp. gr. 1014, acid reaction; moderate amount of albumen; some granular and epithelial casts. Treatment consisted of tinct. Ferri, chlorate, with milk diet, and some farinaceous food, but no meat; exercise in open air as the weather permitted. Urine was examined every week, and some albumen and casts found.

January 4, 1884, ordered tinct. nucis vomica, and spts. nitre, and glycerine, with the muriated tincture of iron.

April 12, albumen in urine very slight, and very few casts; patient has gained several pounds in weight. Ordered 1.20 grain chloride of gold and sodium in glycerine three times a day.

May 1, patient returned to Milwaukee; general appearance improved; more strength; less fatigue on exercising.

November, 1885, she returned to Marietta. Has been very well all summer; has increased in weight; color healthy; urine showed very slight cloudiness of albumen and few pieces of casts. During winter several examinations made, and no albumen or casts found, and patient left for New York last May, well. Dr. E. G. January was consulted on arrival at New York, and pronounced the case cured.

I have thus given this full history, as, to me, the case was an instructive one, first, on account of the severity of the symptoms, and the long continuance of albumen and casts in the urine, and their disappearance at last. I think the happy result was caused by the very careful watching of the patient by her mother, to see that she did nothing to interfere with the treatment, or to cause any relapse. Again, the strict attention given to the milk diet, and avoiding everything which might bring any extra strain on the kidneys. Again, the age of the patient was in her favor. The continued use of the iron and nux vomica tended to prevent anemia and nerve irritation. The case affords another illustration of the *vis medicatrix Naturæ* to overcome disease.

